# Understanding abortion-related complications in health facilities: results from WHO multicountry survey on abortion (MCS-A) across 11 sub-Saharan African countries

**DOI:** 10.1136/bmjgh-2020-003702

**Published:** 2021-01-29

**Authors:** Zahida Qureshi, Hedieh Mehrtash, Seni Kouanda, Sally Griffin, Veronique Filippi, Philip Govule, Soe Soe Thwin, Folasade Adenike Bello, Luis Gadama, Ausbert Thoko Msusa, Nafiou Idi, Sourou Goufodji, Caron Rahn Kim, Jean-Jose Wolomby-Molondo, Kidza Yvonne Mugerwa, Cassimo Bique, Richard Adanu, Bukola Fawole, Thierry Madjadoum, Ahmet Metin Gülmezoglu, Bela Ganatra, Özge Tunçalp

**Affiliations:** 1 Department of Obstetrics and Gynaecology, School of Medicine, University of Nairobi, Nairobi, Kenya; 2 UNDP/UNFPA/UNICEF/WHO/World Bank Special Programme of Research, Development and ResearchTraining in Human Reproduction (HRP), Department of Sexual and Reproductive Health and Research, World Health Organization, Geneva, Switzerland; 3 Institut de Recherche en Science de la Santé, Burkina Faso and Institut africain de Santé Publique, Ouagadougou, Burkina Faso; 4 Centro Internacional Para Saúde Reprodutiva (ICRH-M), Maputo, Mozambique; 5 Department of Infectious Disease Epidemiology, London School of Hygiene & Tropical Medicine, London, UK; 6 Department of Epidemiology and Disease Control, School of Public Health, University of Ghana, Accra, Ghana; 7 Department of Obstetrics and Gynaecology, University of Ibadan, Ibadan, Nigeria; 8 College of Medicine, Department of Obstetrics and Gynaecology, University of Malawi, Zomba, Malawi; 9 Centre for Reproductive Health, College of Medicine, University of Malawi, Blantyre, Malawi; 10 Université Abdou Moumouni de Niamey, Niamey, Niger; 11 Centre de Recherche en Reproduction Humaine et en Démographie (CERRHUD), Cotonou, Benin; 12 Cliniques Universitaires de Kinshasa, Kinshasa, Democratic Republic of Congo; 13 Department of Obstetrics and Gynaecology, Makerere University, Kampala, Uganda; 14 Mozambican Society of Obstetrician and Gynaecologists (AMOG), Maputo, Mozambique; 15 Department of Population, Family and Reproductive Health, University of Ghana School of Public Health, Accra, Ghana; 16 Hopital Regional de Koumra, N’Djamena, Chad

**Keywords:** obstetrics, cross-sectional survey, epidemiology

## Abstract

**Introduction:**

Complications due to unsafe abortions are an important cause of morbidity and mortality in many sub-Saharan African countries. We aimed to characterise abortion-related complication severity, describe their management, and to report women’s experience of abortion care in Africa.

**Methods:**

A cross-sectional study was implemented in 210 health facilities across 11 sub-Saharan African countries. Data were collected on women’s characteristics, clinical information and women’s experience of abortion care (using the audio computer-assisted self-interviewing (ACASI) system). Severity of abortion complications were organised in five hierarchical mutually exclusive categories based on indicators present at assessment. Descriptive bivariate analysis was performed for women’s characteristics, management of complications and reported experiences of abortion care by severity. Generalised linear estimation models were used to assess the association between women’s characteristics and severity of complications.

**Results:**

There were 13 657 women who had an abortion-related complication: 323 (2.4%) women were classified with severe maternal outcomes, 957 (7.0%) had potentially life-threatening complications, 7953 (58.2%) had moderate complications and 4424 (32.4%) women had mild complications. Women who were single, multiparous, presenting ≥13 weeks of gestational age and where expulsion of products of conception occurred prior to arrival to facility were more likely to experience severe complications. For management, the commonly used mechanical methods of uterine evacuation were manual vacuum aspiration (76.9%), followed by dilation and curettage (D&C) (20.1%). Most frequently used uterotonics were oxytocin (50∙9%) and misoprostol (22.7%). Via ACASI, 602 (19.5%) women reported having an induced abortion. Of those, misoprostol was the most commonly reported method (54.3%).

**Conclusion:**

There is a critical need to increase access to and quality of evidence-based safe abortion, postabortion care and to improve understanding around women’s experiences of abortion care.

**Key questions**

**What is already known?**

- According to the most recent estimates, unsafe abortions account for half of all abortions globally, with the majority of the abortion-related deaths occurring in Africa.
- As a result, serious complications arise from unsafe abortions; however, given the lack of use of standard definitions, identification criteria and standardised measurement tools, as well as variations in estimations of the complications, there is limited evidence on the morbidity associated with abortion-related complications.
- As stated at the 1994 International Conference on Population and Development (ICPD) and reiterated in 2019 at ICPD25, it is imperative to reduce abortion-related complications as it is an integral part of sexual and reproductive health and key to reducing maternal mortality.

**What are the new findings?**

- This is one of the few global studies to provide data on abortion-related complications, collecting data across 210 health facilities in 11 sub-Saharan African countries using a standardised tool.
- This study provides insights on the burden and management of abortion-related complications in health facilities using a hierarchal severity gradient, according to sociodemographic, obstetric and clinical characteristics.
- Furthermore, this is the first WHO multicountry survey to explore women’s experience of care during postabortion care.

**Key questions**

**What do the new findings imply?**

- Abortion-related complications are an important underlying and contributing cause of maternal morbidity and mortality as unsafe abortion remains a critical public health issue in sub-Saharan Africa.
- Further efforts are required to increase access to safe abortion and contraception services and improving the quality of postabortion care including the implementation of evidence-based practices and better understanding around women’s experiences of abortion care.

## Introduction

According to the most recent estimates, between 2015 and 2019, 73.3 million abortions occurred worldwide each year, with 8 million abortions occurring in sub-Saharan Africa.^1 2^ Globally, it has been estimated that 45.1% (95% CI 40.6 to 50.1) abortions are unsafe,^1 2^ with75.6% (95% CI 66.4 to 81·4) of abortions occurring in the Africa region classified as unsafe. In the region, almost all unsafe abortions were categorised as least safe, the lowest ranking in the three-tiered approach, defined as abortion provision by untrained individuals using a dangerous method.^1^ The highest proportion of least safe abortions occurred in middle Africa, followed by West Africa and East Africa. To inform better policies and practices related to safe abortion and postabortion care, WHO has been developing guidelines on management of abortion, the role of healthcare workers including the women herself in the case of medical abortion as well as management of abortion-related complications.^3–5^ WHO has recently updated its recommendations, synthesising the latest evidence, to respond to the increased use of medical abortion globally.^6^

Severe abortion-related complications arise from least safe abortions with almost a third (31.3%, 95% CI 21.0 to 41.9) in legally restrictive settings.^1^ These complications are an important and preventable cause of maternal mortality.^7^ Between 2008 and 2013, it was estimated that 9.6% (95% CI 5.1 to 17.2) of maternal deaths are attributable to abortion-related causes in sub-Saharan Africa.^7^ Studies that look at the morbidity from abortion globally and in Africa, using a standard measurement of severity and management of these complications are limited and varied.^8–13^ Current studies offer quantification of the abortion-related complications, but they often do not investigate the gradient or severity of these complications and, do not assess the quality of the care provided.

Since the initial launch and 25th anniversary of the International Conference on Population and Development, it has been stated that abortion care is an integral part of sexual and reproductive health.^14 15^ However, the provision of safe abortion and postabortion care in many countries may be hindered by legally restrictive abortion laws and policies, weak health systems, socioeconomic conditions, the availability of safe abortion services and the stigma surrounding abortion. Capturing accurate information on abortion care can be challenging in such restricted settings. Furthermore, the Lancet Commission on High-Quality Health Systems highlighted that stigmatised conditions such as abortion make individuals more susceptible and vulnerable to poor-quality care.^16^ Women’s experiences of care should be an integral component of high-quality abortion care.^17^

Previous WHO multicountry survey research network studies on maternal and newborn health have been limited in terms of the insight they provided on abortion-related complications.^18–20^ To inform policy and programmatic actions to improve quality of abortion-related care in facilities, in the multicountry survey on abortion-related morbidity (MCS-A), we evaluated the severity of abortion-related complications and the management of these complications, and explored the experience of abortion care reported by women across 11 sub-Saharan African countries.^21^

## Methods

### Study design and participants

The study protocol of the WHO MCS-A study has been published previously, describing the methodology of the cross-sectional study with prospective data collection across health facilities.^21^ This analysis focuses on the primary findings from the sub-Saharan Africa region. Briefly, after a multistage sampling, the 11 participating countries were identified (Benin, Burkina Faso, Chad, Democratic Republic of the Congo (DRC), Ghana, Kenya, Malawi, Mozambique, Niger, Nigeria, Uganda), followed by provinces and facilities in each country.^22^

Health facilities were only eligible if they fulfilled the following characteristics: >1000 deliveries per year, a gynaecology ward and surgical capability (defined as providing the signal functions for comprehensive emergency obstetric care, which includes removal of retained products and surgical capability^23^ and, if available, abortion provision and/or postabortion care. To ensure each facility could contribute sufficient data to the study during the 3-month data collection period, facilities reporting <10 postabortion care patients on average over a month in the facility assessment form were excluded. In each country, data collection took place over a 3-month period between February 2017 and April 2018.

All women presenting to the participating facilities with signs and symptoms or death at discharge from abortion-related complications or early pregnancy loss (including ectopic and molar pregnancies) were included. Pregnant women with a diagnosis of threatened abortion, defined as vaginal bleeding with a closed cervix were excluded.^24^ The criterion of all women presenting to the facilities rather than admission to the facility was used to avoid exclusion of those women who seek care in the facilities for mild complications. Women with abortion-related complications who were admitted or had a prolonged hospital stay (>24 hours), able and were willing to consent were eligible to participate in the exit survey and convenience sampling was used to invite eligible women to participate in the exit survey based on the workload of data collectors and time of day in the study facility.

### Procedures

A hospital administrator or a healthcare provider responsible for the gynaecology and obstetrics wards at each identified facility completed the facility assessment form that collected information on availability of services and resources. For the main survey, 1-week training sessions were conducted with research assistants at the facility and country level on the objectives of the study, data collection procedures, practice sessions with the tools as well as highlighting ethical, safety and confidentiality considerations. Techniques of information gathering on this sensitive and highly stigmatised topic using the medical records were also conducted during the training session and data collectors had access to facility coordinators and principal investigators for continuous support. Based on the eligibility criteria, research assistants at each facility reviewed and abstracted information from women’s medical records that included sociodemographic data, clinical information, obstetrics characteristics, signs and symptoms due to abortion-related complication, medical procedures, clinical outcomes and vital status at discharge to identify eligible women. Abstracted medical records data were transcribed into paper-based case report forms and entered into a web-based electronic data capture system developed by the Centro Rosario de Estudios Perinatales (Rosario, Argentina) for the study. Data entry was performed at the health facility or at a central level, dependent on logistics and available infrastructure. For the exit survey, data were collected on tablets using the audio computer-assisted self-interviewing (ACASI) system developed by Tufts University. The system allowed participants to respond to the exit survey at a private location with a focus on maintaining participant confidentiality. Data collected in the exit survey consisted of abortion safety characteristics (method used, provider, setting) prior to coming to the facility, and women’s experience of abortion care related to effective communication, respect and dignity and emotional support during their time in the facility. The women who participated in the exit survey were compensated for their time by approximately US$2 worth of mobile phone airtime. Data managers in Argentina continuously monitored the study data flow and data quality by use of validation procedures and progress reports for all countries. Data inconsistencies were identified and corrected by contacting the study principal investigators as they emerged. These procedures have been used in previous multicentre studies.^25^

### Description of measures

**Figure 1:**
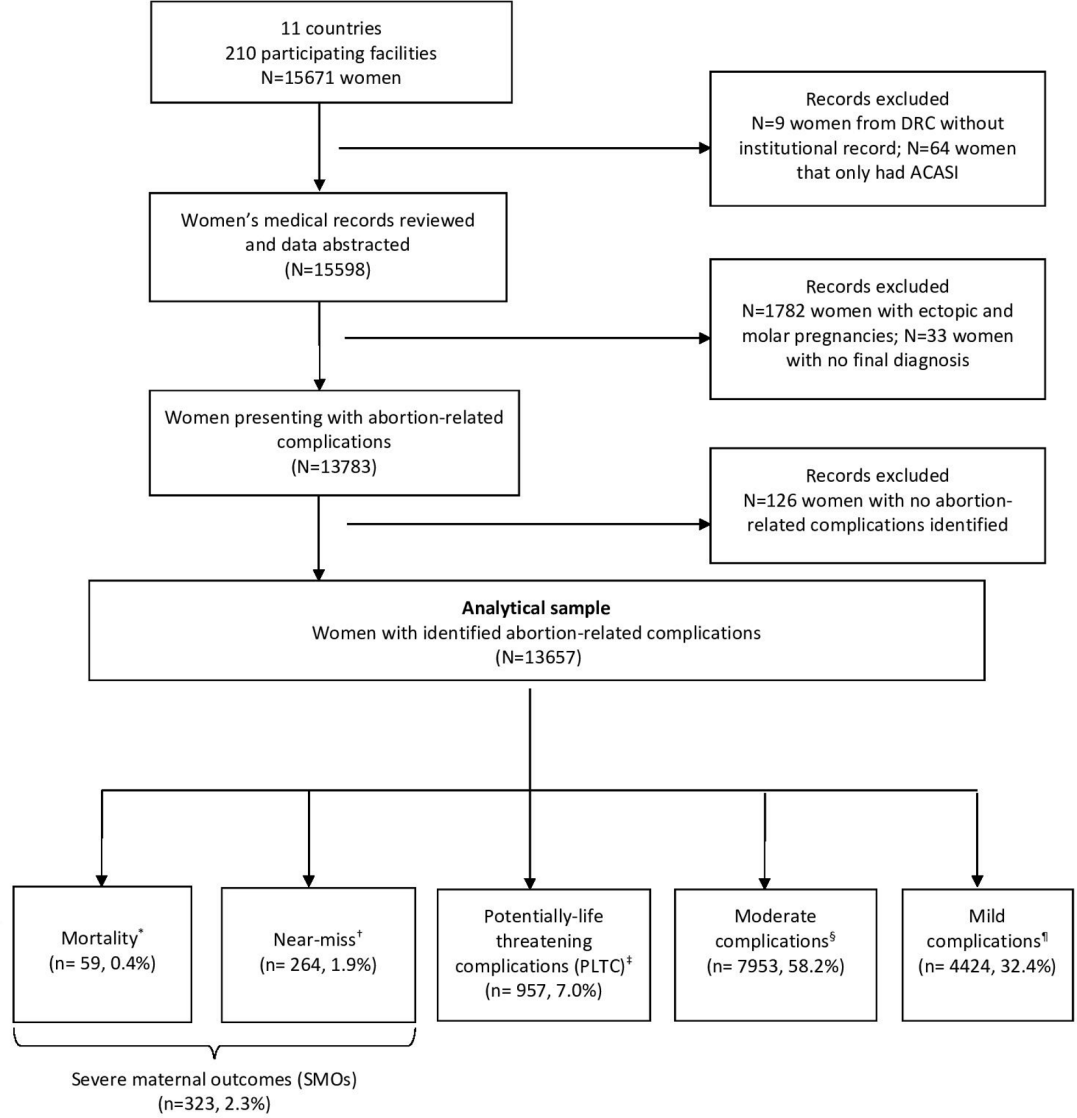
Study flow diagram for severity of abortion-related complications. Severe maternal outcomes (SMOs) (n=323, 2.3%). *Status at discharge. †WHO maternal near-miss criteria (organ dysfunction of either one or more of the following: cardiovascular, respiratory, renal, coagulation, hepatic, neurological or uterine dysfunction). ‡WHO potentially life-threatening complications (severe haemorrhage, severe systemic infection or suspected uterine perforation). §Moderate complications (heavy bleeding, suspected intra-abdominal injury or infection). ¶Mild complications based on abnormal physical examination findings on initial assessment (vital signs, appearance, mental status, abdominal examination, gynaecological examination).

Based on indicators present at time of hospital admission including clinical, laboratory and management-based markers, abortion-related complications were classified into five hierarchical and mutually exclusive categories based on severity: (1) *deaths*, (2) *near miss*, (3) *potentially life-threatening complications*, (4) *moderate complications* and (5) *mild complications* (figure 1).

Based on WHO criteria for near miss, women who died or identified as a near-miss case were classified as a *severe maternal outcome*.^26^ Women presenting with severe haemorrhage, severe systemic infection or suspected uterine perforation were classified based on WHO’s criteria for *potentially life-threatening conditions*.^26^ *Moderate complications* included bleeding, suspected intra-abdominal injury and infection. *Mild complications* included any abnormal signs from initial physical examination (vital signs, appearance, mental status, abdominal examination, gynaecological examination). Death was based on woman’s status at discharge. Online supplemental file 4 includes the identification criteria used for each severity category in detail and, online supplemental file 1 includes information on timing of abortion-related complications based on facility admission.

10.1136/bmjgh-2020-003702.supp4 Supplementary data

10.1136/bmjgh-2020-003702.supp1 Supplementary data

Gestational age at presentation was grouped as <13 weeks, ≥13 weeks or undetermined weeks (online supplemental file 1). Clinical management of abortion-related complications was categorised as medically managed by uterotonics only, by uterine evacuation only or both methods. Uterotonics use was further divided into: misoprostol alone, oxytocin alone, ergometrine only and their combinations. Uterine evacuation was further examined by type of procedure: manual vacuum aspiration (MVA), dilation and curettage (D&C) and both.

### Patient and public involvement

Development of the protocol used evidence from qualitative research exploring women’s experiences with abortion care. The been published, the results will be disseminated for professional and non-professional audiences in participating countries.

### Data analysis

Descriptive bivariate analysis was performed for national level and facility level characteristics (online supplemental file 1), as well as sociodemographic, obstetrics and clinical management characteristics by severity of abortion-related complications. The χ^2^ test was used to compare proportions of descriptive characteristics across severity categories. Severity of abortion-related complications is presented across countries as ratios calculated based on the prevalence of each category of complication per 1000 women with complications.

Descriptive analysis was also performed to evaluate the methods used, information received and help sought to end pregnancy for self-reported data collected in the exit interview via the ACASI platform. Experience of abortion care during facility stay was assessed by comparing responses across severity of abortion-related complications using χ^2^ test.

Regression methods were used to evaluate women’s characteristics potentially associated with the outcome of abortion-related complication severity. Generalised linear models, adjusting for facility clustering effect and differences across countries, were fitted to estimate the odds of severe maternal outcomes, potentially life-threatening complications and, moderate complications for women’s characteristics. The independent variables were categorical variables including sociodemographic characteristics (age, marital status, education, gainful occupation) and, obstetric characteristics (prior pregnancies, gestational age and expulsion of products of conception before arrival to the health facility).

Data analysis was conducted using SAS (V.9.4, Cary, North Carolina, USA).

## Results

We collected data on 15 671 women attending 210 facilities in 11 sub-Saharan African countries (figure 1); 124/210 (59∙1%) of the facilities were secondary level facilities and most included received care in urban facilities (9,839, 72%). Details on facility characteristics included can be found in the online supplemental file 3.

10.1136/bmjgh-2020-003702.supp3 Supplementary data

A total of 73 women were excluded, of which 9 were excluded from these analyses as they did not have corresponding facility records and 64 had ACASI data only without corresponding individual records. A further 1815 (13∙2%) women were excluded because they had complications related to molar and ectopic pregnancies (n=1782) or they did not have a final diagnosis (n=33). For 126 (0∙9%) women, there was insufficient information collected for severity to be determined and these women were excluded from the analyses. The final sample included 13 657 medical records and the analysis of these records focused on women with abortion-related complications.

Based on the inclusion criteria and available data on admission and discharge dates, 65.6% (8931/13614) of the women with abortion-related complications were eligible for ACASI exit interview. Of those, a total of 3091/8931 (34∙6%) women were recruited and participated in the ACASI exit interview.

Table 1 compares national, facility, demographic and obstetric characteristics of women, according to complication severity. Nearly half the sample, 6493 (47∙6%) were from countries where both misoprostol and mifepristone were registered for medical abortion, and 7435 women (54∙4%) were from countries which do not have any national guidelines on abortion (induced abortion or postabortion care). Online supplemental file 2 describes the legal status of abortion in the study countries, with the majority of countries having a law that allows or permits abortion only on one or more legal grounds.^27^

10.1136/bmjgh-2020-003702.supp2 Supplementary data

**Table 1:** National, sociodemographic and obstetric characteristics of study population by severity of abortion-related complications

	Total (n=13 657)	Severe maternal outcomes (n=323)	Potentially life-threatening complications (n=957)	Moderate (n=7953)	Mild (n=4424)
National					
Country-recognised medical abortion*,†					
None	2980 (21.8)	83 (25.7)	158 (16.5)	1919 (24.1)	820 (18.5)
Misoprostol	4183 (30.6)	123 (38.1)	263 (27.5)	2098 (49.5)	1699 (38.4)
Misoprostol-Mifepristone	6493 (47.6)	117 (36.2)	536 (56.0)	3936 (49.5)	1905 (43.1)
National guidelines on abortions*,‡					
None	7435 (54.4)	186 (57.6)	480 (50.2)	4242 (53.3)	2527 (57.1)
Postabortion care only	2429 (17.8)	33 (10.2)	236 (24.7)	1509 (18.9)	651 (14.7)
Induced abortion and postabortion care	3793 (27.8)	104 (32.2)	241 (25.2)	2202 (27.7)	1246 (28.2)
Facility					
Facility type*					
Primary	1022 (8.4)	31 (9.9)	76 (8.7)	748 (10.4)	167 (4.4)
Secondary	7505 (61.7)	139 (44.6)	421 (48.1)	4408 (61.4)	2537 (66.9)
Tertiary	3630 (29.8)	142 (45.5)	378 (43.2)	2027 (28.2)	1083 (28.6)
Location*					
Urban	9839 (72.0)	262 (81.1)	651 (68.0)	5717 (71.9)	3209 (72.5)
Peri-urban	2080 (15.2)	32 (9.9)	105 (10.9)	1206 (15.2)	737 (16.6)
Rural	1738 (12.7)	29 (8.9)	201 (21.0)	1030 (12.9)	478 (10.8)
Sociodemographic and obstetric					
Age (in years)	13 516				
≤19	2173 (16.1)	58 (18.1)	157 (16.5)	1222 (15.5)	736 (16.9)
20–29	6565 (48.6)	151 (47.0)	451 (47.3)	3866 (49.1)	2097 (48.1)
≥30	4778 (35.4)	112 (34.9)	345 (36.2)	2791 (35.4)	1530 (35.1)
Marital status*	12 614				
Single	2852 (22.6)	104 (34.3)	246 (27.9)	1628 (22.2)	874 (21.4)
Married/Cohabitating	9474 (69.4)	186 (61.6)	609 (69.1)	5548 (75.5)	3131 (76.7)
Separated/Divorced/Widowed	288 (2.3)	12 (3.9)	27 (3.1)	174 (2.4)	75 (1.8)
Education*	10 485				
No education	1791 (17.1)	41 (17.1)	125 (17.1)	1095 (18.3)	530 (15.0)
Primary	3004 (28.7)	63 (26.3)	245 (33.6)	1640 (27.4)	1056 (29.9)
Secondary or more	5690 (54.3)	136 (56.7)	360 (49.3)	3252 (54.3)	1942 (55.1)
Gainful occupation*	11 708				
Yes	5276 (45.1)	119 (43.1)	337 (41.0)	3302 (48.4)	1518 (40.1)
Previous pregnancies	13 264				
0	3584 (27.0)	79 (24.8)	231 (24.6)	2090 (26.9)	1184 (27.9)
1 or more	9680 (72.9)	240 (75.2)	708 (75.4)	5678 (73.1)	2054 (72.1)
Previous abortions	9434				
0	6050 (64.1)	154 (65.5)	462 (66.9)	3493 (63.1)	1941 (65.3)
1 or more	3384 (35.9)	81 (34.5)	229 (33.1)	2041 (36.9)	1033 (34.7)
Gestational age (in weeks)*					
<13	7214 (52.8)	118 (36.5)	378 (39.5)	4284 (53.9)	2434 (55.0)
13–28	3998 (29.3)	112 (34.7)	372 (38.9)	2211 (27.8)	1303 (29.5)
Undetermined	2445 (17.9)	93 (28.8)	207 (21.6)	1458 (18.3)	687 (15.5)
Expulsion of products of conception before arrival to facility*	13 616				
Yes	5916 (43.5)	177 (55.1)	511 (53.7)	3299 (41.6)	1929 (43.7)

*P<0.0001.

†None (Niger, Burkina Faso, Chad); misoprostol (Benin, Ghana, Kenya, Uganda); misoprostol-mifepristone (DRC, Malawi, Mozambique, Nigeria).

‡None (Burkina Faso, DRC, Chad, Malawi, Niger, Uganda); postabortion care (Kenya only); induced abortion and postabortion care (Benin, Ghana, Mozambique, Nigeria).

DRC, Democratic Republic of the Congo.

### Severity of abortion-related complications

Of all the women who had abortion-related complications (n=13 657), 264 (1∙9%) women were classified as near-miss cases, 957 (7∙0%) had potentially life-threatening complications, 7953 (58∙2%) had moderate complications and 4424 (32∙4%) women had mild complications. In addition, there were 59 (0∙4%) deaths due to abortion-related complications. Abortion-related deaths and women with near-miss complications were grouped as severe maternal outcomes, representing a total of 323 women (2∙6%) (figure 1). Among women with near miss, the most common organ dysfunction identified was cardiovascular (165/264, 62∙5%). Among potentially life-threatening, moderate and mild complications, the most commonly identified complications were severe haemorrhage (703/957, 73∙2%), heavy bright bleeding (7656/7953, 96∙3%) and vaginal bleeding (3470/4424, 78∙4%) (online supplemental file 4).

Among women presenting to facilities with an abortion-related complication (n=13 657), they were between the ages of 20 and 29 years, and most were married or cohabitating (77∙4%) and reported secondary education or above (54∙3%). Less than half of the women were gainfully employed. According to their obstetric history, most women had a previous pregnancy (91∙5%) and many (64∙1%) did not have any previous abortions. Significant differences were observed across severity categories for marital status, education, gainful occupation and timing of expulsion of products of conception.

**Figure 2:**
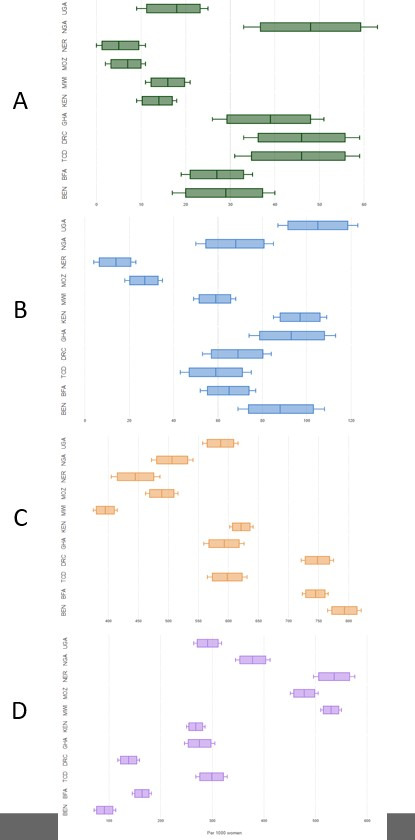
The severity of abortion-related complications across countries per 1000 women. A: Severe maternal outcomes; B: Potentially-life threatening complications; C: Moderate complications; D: Mild complications. Figures are drawn to scale for each severity category. BEN, Benin; BFA, Burkina Faso; TCD, Chad; DRC, Democratic Republic of Congo; GHA, Ghana; KEN, Kenya; MWI, Malawi; MOZ, Mozambique; NER, Niger; NGA, Nigeria; UGA, Uganda

Figure 2 describes the severity of complications across countries per 1000 women with complications. Severe maternal outcomes ranged from 5 per 1000 women in Niger to 48 per 1000 women in Nigeria. Potentially life-threatening complications ranged from 14 per 1000 women in Niger to 105 per 1000 women in Uganda. Moderate complications ranged from 395 per 1000 women in Malawi to 795 per 1000 women in Benin, and the mild complications ranged from 91 per 1000 women in Benin to 536 per 1000 women in Niger.

Table 2 presents generalised linear models evaluating women’s characteristics associated with severe maternal outcomes, potentially life-threatening and moderate abortion-related complications compared with mild complications, adjusting for country, age, education, occupation, prior pregnancy and gestational age and timing of expulsion of products of conception. In the adjusted model, being single (adjusted OR (aOR): 3∙4 (95% CI 2∙2 to 5∙3)), having prior pregnancies (aOR: 2∙1 (95% CI 1∙3 to 3∙5)) and presenting at later gestational ages (≥13 weeks) (aOR: 3∙5 (95% CI 2∙3 to 5∙4)) were positively associated with experiencing severe maternal outcomes compared with women with mild complications. Furthermore, our model shows that women who had expulsion of products of conception prior to arrival to the health facility were twice (aOR: 2∙3 (95% CI 1∙6 to 3∙4)) as likely to experience severe maternal outcomes.

**Table 2:** Determinants of increased risk of abortion-related complication severity compared with mild complications observed among women who sought care at a facility

		SMOs vs mild		PLTC vs mild		Moderate vs mild	
		aOR	95% CI	aOR	95% CI	aOR	95% CI
Age (in years)	≤19	1.5	(0.8 to 2.7)	1.0	(0.8 to 1.3)	1.1	(0.9 to 1.3)
	20–29	0.9	(0.7 to 1.2)	0.9	(0.8 to 1.1)	1.1	(0.9 to 1.2)
	≥30	Reference	Reference	Reference	Reference	Reference	Reference
Marital status	Single	3.4†	(2.2 to 5.3)	1.5†	(1.2 to 1.8)	1.0	(0.9 to 1.1)
	Other than single	Reference	Reference	Reference	Reference	Reference	Reference
Education	No education	0.7	(0.4 to 1.3)	1.5*	(1.2 to 1.9)	1.0	(0.8 to 1.2)
	Primary	1.3	(0.9 to 1.9)	1.4†	(1.2 to 1.7)	1.1*	(1.0 to 1.3)
	Secondary or more	Reference	Reference	Reference	Reference	Reference	Reference
Gainful occupation	No	1.2	(0.8 to 1.6)	1.1	(0.9 to 1.3)	0.9	(0.9 to 1.1)
	Yes	Reference	Reference	Reference	Reference	Reference	Reference
Prior pregnancies	1 or more	2.1*	(1.3 to 3.5)	1.2*	(1.0 to 1.5)	1.1	(0.9 to 1.2)
	0	Reference	Reference	Reference	Reference	Reference	Reference
Gestational age (in weeks)	13–28	3.5†	(2.3 to 5.4)	1.7*	(1.3 to 2.2)	1.1	(0.9 to 1.4)
	Undetermined	2.3†	(1.6 to 3.2)	1.4*	(1.2 to 1.6)	1.1	(0.9 to 1.2)
	<13 weeks	Reference	Reference	Reference	Reference	Reference	Reference
Expulsion of products of conception before arrival to facility*	Yes	2.3†	(1.6 to 3.4)	1.8†	(1.4 to 2.2)	1.0	(0.9 to 1.2)
	No	Reference	Reference	Reference	Reference	Reference	Reference

*P<0.05.

†P<0.0001.

aOR, adjusted OR; PLTC, potentially life-threatening complication; SMO, severe maternal outcome.

When women with potentially life-threatening complications were compared with mild complications (adjusting for country, age, education, occupation, prior pregnancy and gestational age and timing of expulsion of products of conception), similar pattern to severe maternal outcomes were observed (table 2). In the adjusted model, being single (aOR: 1∙5 (95% CI 1∙2 to 1∙8)), having prior pregnancies (aOR: 1∙2 (95% CI 1∙0 to 1∙5)), presenting at later gestational ages (≥13 weeks) (aOR: 1∙7 (95% CI 1∙3 to 2∙2)) and expulsion of products of conception prior to arrival to the health facility (aOR: 1∙8 (95% CI 1∙4 to 2∙2)) are positively associated with experiencing potentially life-threatening complications. Women with no or primary education were more likely to experience potentially life-threatening complications: 1∙5 (95% CI 1∙2 to 1∙9), 1∙4 (95% CI 1∙2 to 1∙7). Finally, women with moderate complications had no significant associations, except slightly elevated odds with primary education 1∙1 (95% CI 1∙0 to 1∙3), compared with mild complications.

### Management of abortion-related complications

As shown in table 3, approximately 50% of the women received both uterotonics and underwent uterine evacuation for management of abortion-related complications across all severity categories. For other management types, 16∙7% of women received uterotonics only and 27∙6% of women underwent uterine evacuation only. Across severity categories, for mild complications, uterine evacuation was performed more commonly (35∙6%) than for the other groups. Women with potentially life-threatening (56∙7%) or moderate complications (52∙5%) more commonly received both uterotonics and uterine evacuation compared with women with mild complications (44∙2%) and severe maternal outcomes (35∙3%).

**Table 3:** Uterotonics and uterine evacuation for management of abortion-related complications by severity*

		Severity of abortion-related complications				
Management of complications	Total (n=13 657)	Severe maternal outcomes (n=323)	Potentially life-threatening complications (n=957)	Moderate (n=7953)	Mild (n=4424)	P value
Uterotonics	2280 (16.7)	62 (19.2)	120 (12.5)	1493 (18.8)	605 (13.7)	<0.0001
Uterine evacuation	3765 (27.6)	74 (22.9)	212 (22.2)	1904 (23.9)	1575 (35.6)	
Both uterotonics and uterine evacuation	6790 (49.7)	114 (35.3)	543 (56.7)	4177 (52.5)	1956 (44.2)	
Other	634 (4.6)	68 (21.1)	77 (8.1)	282 (3.6)	207 (4.7)	
None	188 (1.4)	5 (1.6)	5 (0.52)	97 (1.2)	81 (1.8)	

*Mutually exclusive.

Table 4 details the type of management for our study sample. Overall, 66∙4% of women received uterotonics, and among those the most commonly used was oxytocin (50∙9%), followed by misoprostol (22∙7%). Uterine evacuation was performed in 77∙3% women, the most common method used was MVA (in 76∙9% women), and its use was most frequent in the moderate cases (79∙3%). Following MVA, D&C was used among 20∙1% of women. 8∙1% of the women received blood products and their use was mostly among the severe maternal outcomes (55∙4%) and potentially life-threatening complications (48∙2%). A total of 121 women underwent major surgeries. The most common were exploratory laparotomy, (76%) and hysterectomy (21∙5%). Almost 9 out of 10 women received antibiotics for prophylaxis of treatment. Among the small number of women (77, 0∙6%) who were admitted to intensive care unit, the majority were those with severe maternal outcomes.

**Table 4:** Types of management by severity of abortion-related complications†

		Severity of abortion-related complications			
	Total (n=13 657)	Severe maternal outcomes (n=323)	Potentially life-threatening complications (n=957)	Moderate (n=7953)	Mild (n=4424)
Uterotonics*	9071 (66.4)	176 (54.5)	663 (69.3)	5670 (71.3)	2562 (57.9)
Misoprostol only	2055 (22.7)	33 (18.8)	117 (17.7)	1293 (22.8)	612 (23.9)
Misoprostol and ergometrine	154 (1.7)	0	6 (0.9)	110 (1.9)	38 (1.5)
Oxytocin only	4622 (50.9)	94 (53.4)	353 (53.2)	2749 (48.5)	1426 (55.7)
Oxytocin and ergometrine	463 (5.1)	3 (1.7)	40 (6.0)	392 (6.9)	28 (1.1)
Ergometrine	346 (3.8)	7 (3.9)	26 (3.9)	192 (3.4)	121 (4.7)
Misoprostol and oxytocin	1102 (12.2)	30 (17.1)	98 (14.8)	673 (11.9)	301 (11.8)
Misoprostol, oxytocin and ergometrine	291 (3.2)	9 (5.1)	22 (3.3)	230 (4.1)	30 (1.2)
Other	38 (0.4)	0	1 (0.2)	31 (0.6)	6 (0.2)
Uterine evacuation*	10 555 (77.3)	188 (58.2)	755 (78.9)	6081 (76.5)	3531 (79.9)
Manual vacuum aspiration (MVA)	8124 (76.9)	136 (72.3)	544 (72.1)	4823 (79.3)	2621 (74.2)
Dilation and curettage (D&C) Only	2125 (20.1)	48 (25.5)	185 (24.5)	1065 (17.5)	827 (23.4)
Both MVA and D&C	56 (0.5)	1 (0.5)	8 (1.1)	36 (0.6)	11 (0.3)
Other	250 (2.4)	3 (1.6)	18 (2.4)	157 (2.6)	72 (2.0)
Blood transfusion*	1129 (8.3)	179 (55.4)	461 (48.2)	368 (4.6)	121 (2.7)
1 unit	570 (51.6)	65 (36.9)	218 (47.6)	214 (60.8)	73 (61.9)
2 units	365 (33.1)	62 (35.2)	171 (37.3)	96 (27.3)	36 (30.5)
3 units or more	169 (15.3)	49 (27.8)	69 (15.1)	42 (11.9)	9 (7.6)
Surgical procedures*	121 (0.88)	40 (12.4)	41 (4.3)	19 (0.24)	21 (0.47)
Laparoscopy	3 (2.5)	0	0	3 (15.8)	0
Exploratory laparotomy	92 (76.0)	24 (60.0)	40 (97.6)	14 (73.7)	14 (66.7)
Hysterectomy	26 (21.5)	16 (40.0)	1 (2.4)	2 (10.5)	7 (33.3)
Antibiotics received for prophylaxis or treatment*	12 215 (89.5)	6516 (90.3)	3576 (89.4)	7205 (90.6)	3794 (85.8)
Admission to intensive care unit*	77 (0.6)	38 (11.8)	20 (2.1)	10 (0.1)	9 (0.2)

*P<0.0001.

†Not mutually exclusive.

### Women’s self-reported experiences of abortion care via ACASI exit survey

Distribution of sociodemographic and obstetric characteristics of the women who participated in the ACASI exit interview were not significantly different from the overall study population (except for gestational age) (online supplemental file 5). Of the 3091 women who undertook ACASI exit interview, only 602 women (19∙5%) reported having used one method or more to end their pregnancy. The most commonly reported methods to end the pregnancy were: misoprostol (54∙3%), other medicines either orally or vaginally (40∙5%) and procedures that cleared out contents from the uterus (38∙7%). Furthermore, 18∙7% reported using of herbs, antimalarial drugs, bleach, gasoline and detergents and 14∙3% reported using traditional abdominal massage.

10.1136/bmjgh-2020-003702.supp5 Supplementary data

**Figure 3:**
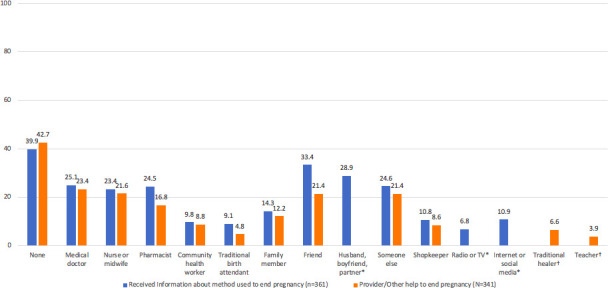
Self-reported sources of information and help used to end pregnancy (n=602). *Response option only included for information question. †Response option only included for help question.

Of the 602 women who reported induced abortion, 241 (39∙9%) reported that they did not receive any information about the method used to end pregnancy. For the rest, the most commonly reported sources of information were friends (33∙4%), husband /partner/boyfriend (28∙9%), followed by healthcare providers (25∙1% medical doctor, 24∙5% pharmacist, 23∙4% nurse/midwife). More than 1 in 10 women reported getting information through internet or social media, whereas only 6∙8% reported radio or TV as a source of information (figure 3). In terms of receiving assistance from someone to end their pregnancy, 261 (42∙7%) of the women did not get any help. The most commonly reported assistance was from healthcare providers (23∙4% medical doctors, 21∙6% nurse/midwifes, 16∙8% pharmacist). Of note, more than one in five women reported being assisted by a friend (figure 3).

Table 5 presents experience of abortion care during facility stay as reported by women. Overall, 19∙1% of the women stated that they were not given explanations regarding their care and treatment. One out of three women reported that they were not able to ask questions during their examination and treatment. Overall, 18∙5% of women felt their choices and preferences were not followed during hospital stay and this differed significantly across severity categories. Women who reported not being spoken to nicely during their stay ranged between 6∙9% among women with potentially life-threatening complications and 9∙8% among women with moderate complications; 13∙2% of women reported not having received pain medications during their stay ranging from 9∙9% among women with severe complications to 14∙6% among women with moderate complications.

**Table 5:** Self-reported experience of abortion care during facility stay

		Severity of abortion-related complications			
	Total (n=3091)	Severe maternal outcomes (n=96)	Potentially life-threatening complications (n=316)	Moderate (n=1851)	Mild (n=828)
		N (%)	N (%)	N (%)	N (%)
Explanations regarding care and treatment (n=2950)					
No	562 (19.1)	25 (27.5)	63 (20.7)	333 (18.9)	141 (17.7)
Able to ask questions during the examination and treatment (n=2950)					
No	1009 (34.2)	31 (34.1)	115 (37.8)	610 (34.7)	253 (31.8)
Feel healthcare provider informed you about decisions taken for care (n=2944)					
No	639 (21.7)	22 (24.2)	73 (24.0)	385 (21.9)	159 (20.0)
Encountered anxiety or stress during hospital stay (n=2942)					
Yes	1587 (53.9)	53 (58.2)	163 (53.9)	949 (54.1)	422 (53.1)
If yes to above (n=1585), not able to tell healthcare provider who helped you that you were feeling anxious or stress	679 (42.8)	20 (37.7)	63 (38.7)	422 (44.6)	174 (41.2)
If yes to above (n=906), not offered additional support when you told the healthcare provider about feeling the anxiety or stress	102 (11.3)	2 (6.1)	6 (6.0)	69 (13.1)	25 (10.1)
Feel choices and preferences were followed during hospital stay* (n=2935)					
No	542 (18.5)	17 (18.7)	48 (16.0)	352 (20.1)	125 (15.7)
Spoken to nicely (n=2940)					
No	257 (8.7)	7 (7.7)	21 (6.9)	172 (9.8)	57 (7.2)
Receive pain medications during hospital stay (n=2940)					
No	389 (13.2)	8 (8.8)	31 (10.3)	255 (14.6)	95 (11.9)
If yes (n=2551), pain medications did not help ease pain	144 (5.6)	5 (6.0)	9 (3.3)	83 (5.5)	47 (6.7)

*P<0.05.

## Discussion

Our study quantified the health burden experienced by women presenting with abortion-related complications in 11 sub-Saharan African countries. We used a gradient to examine and report on the severity of and management of abortion-related complications across 13 657 women, 210 health facilities and subsample of women-reported experiences. Of all women with abortion-related complications, approximately 2.3% were identified as severe maternal outcomes, 7.0% potentially life-threatening complications, 58% moderate and 32% mild complications. The proportion of women presenting with severe maternal outcomes and potentially life-threatening complications indicates that unsafe abortion continues to pose a major public health challenge in Africa. The prevalence of these severe complications varies across countries, compared with other findings, illustrating the issues with comparability due to lack of a standardised approach in measurement of abortion-related complications. Globally, at least 9% of abortion-related complications in health facilities were identified as a near-miss event,^13^ while studies from Africa report varying prevalence of severe abortion-related complications. These studies have used different criteria, ranging from Rees *et al*^28^ to expanded WHO near-miss criteria,^29^ to define and measure complication severity, leading to different estimations as seen from various studies in DRC (16%), Kenya (37%), Malawi (21%), Zambia (16%) and Zimbabwe (19%).^8 9 11 30 31^ Our results underline the importance of using standardised definitions including the application of WHO’s near-miss criteria^29^ to ensure comparability when quantifying the burden of abortion-related complications as well as measuring the progress globally and in the context of Africa.^12 13^

Our results show that 9 out of 10 women who seek care in the facilities have moderate or mild complications (most commonly with some form of vaginal bleeding), which may reflect the changing tide of abortion due to medical abortion. After medical abortion use, WHO recommendations on medical abortion highlights that vaginal bleeding for 2 weeks is normal and serious side effects including adverse events are rarely reported with the appropriate medical abortion regimen.^6^ We were not able to discern whether vaginal bleeding was due to medical abortion use prior to seeking care in facilities. A plausible explanation is that women presenting with mild complications may have been experiencing this common side effect of medical abortion use which highlights the importance of access to quality information and services regarding medical abortion.

Severe abortion-related complications were associated with being single, having a prior pregnancy and late gestational age (≥13 weeks), with over a twofold increase in odds among severe maternal outcomes. These findings are consistent with other studies that found increased likelihood of severe abortion-related complications due to similar characteristics.^8 11 31 32^ Abortion remains a stigmatised issue in Africa creating further inequities and vulnerabilities for women based on their socioeconomic characteristics, such as marital status, contributing to severe complications.^10^ Severe abortion-related complications were also associated with expulsion of products of conception (POC) prior to arrival to the facility. This may be attributable to a number of possible gaps in the information channels regarding the recommended medical abortion dosage, on when to seek postabortion care, side effects and symptoms of possible complications.^10 33–35^ Women might also not be aware of the services available, may fear stigma and mistreatment and use methods which are considered unsafe. According to a systematic review, awareness and knowledge of the abortion laws and policy environment among women was limited, even in countries where the laws were liberal, hindering women from accessing available services.^36^

In terms of management of abortion-related complications at the health facility, approximately half of the abortion-related complications were treated with both uterotonics and uterine evacuation. The use of both uterotonic and uterine evacuation may be a reflection of overmedicalization due to possible provider preference and practice rather than evidence-based recommendations.^37^ This is particularly important among mild complications presenting with vaginal bleeding that may have been due to medical abortion use prior to the facility. The management of abortion-related complications using safe and low-cost technologies, such as MVA, which is the recommended method of uterine evacuation regardless of severity,^3^ is on the rise across countries in our study; however, the use of non-recommended and unsafe methods such as D&C still persists. This may be due to lack of resources (eg, equipment and supplies)^38^ and/or training, which might lead providers to use outdated methods like D&C.^10 12 39^ Furthermore, half of our cases received oxytocin as the most common method of uterotonics, rather than misoprostol, which is recommended to manage abortion-related complications.^6 40^ The less frequent use of misoprostol may be due to lack of availability, familiarity, training and guidelines as well as providers’ perceptions on misoprostol being a less effective and safe method of postabortion care.^12 40^ The inequities in the provision of good quality care highlight the need to strengthen the adoption and implementation of evidence-based recommendations for the provision of postabortion care.^4 41^

Of the participating women in the ACASI exit survey, one in two reported induced abortion with the most commonly used method being misoprostol, whereas one in five still reported using dangerous methods such as antimalarial drugs, or insertion of something into the vagina. This supports the trends observed that medical abortion is becoming more commonly used among women and possibly contributing to the reduction in the burden of severe complications.^1^ Furthermore, our results indicate that a small but significant proportion of women accessed information through social media and internet. This suggests that these channels may be used for informing women about safe abortion options and postabortion care (in areas where internet access is available) paving the way for telemedicine to be adopted given the evidence that it is becoming more acceptable to women and providers.^42^ Reducing the burden of both severe and mild complications among women can be improved through implementation of evidence-based practices which include provision of accurate information and resources for women seeking medical abortion outside of healthcare facilities.^3 33 34 43^ These approaches might also be applicable to better inform women about what to expect after medical abortion and when to seek care from facilities, reducing the burden on women and health systems alike.

For women’s experiences of postabortion care, among those participating in the exit survey, one in five women felt that their choices and preferences were not followed during care and the same proportion did not receive explanations regarding their care. Evidence shows that mistreatment during childbirth in the facility may be a significant barrier to women’s decision to seek care.^44^ Furthermore, provider perceptions on abortion care and workload can negatively impact provider-patient interaction, timeliness and quality of care.^12^ Globally, there is growing interest in measuring person-centred reproductive healthcare including abortion care, contraception and maternity care.^38 45–47^ While the evidence around measuring women’s experiences seeking abortion-related care, in particular, is limited, research efforts are increasing to support the development of measurement tools.^38^

### Strengths and limitations

We sought to use a standardised approach to identify abortion-related complications across 210 facilities in 11 countries. The large sample size and number of participating facilities enables generalisability of results to facilities with similar outcomes and geographical areas similar to those included in our study, and other sub-Saharan African countries (lusophone, francophone, anglophone). Our results are not representative of the population and despite the implementation of standardised definitions for abortion-related complications, a few data collection issues linked to the quality of the medical records (eg, poor record keeping, especially on abortion) may have led to underestimation and/or misclassification of severity across countries. Furthermore, depending on the countries and the context, including the abortion rate, the number percentage of women experiencing a complication after abortion and further seeking care at the facilities might vary. Due to the sensitive nature of the topic in all the study countries, we did not differentiate between induced and spontaneous abortion during data collection, and it should be noted that induced abortion-related complications may be more severe compared with spontaneous abortions.^13^ Our study is based on a cross-sectional survey across facilities and limited to the women who present at these facilities for abortion-related complications. The study also incorporated a confidential self-reported exit interview component to measure women’s experiences of abortion care (prior to coming and during their facility stay) using the ACASI software. ACASI was used given that it is well-suited for collecting data on sensitive data; however, women may have still under-reported induced abortion.

## Conclusion

Unsafe abortion remains a critical public health issue in sub-Saharan African countries, causing significant morbidity for women and burden on health systems. Based on our results, future areas of work include using the standardised approach to quantify abortion-related complications in health facilities, implementing clinical management practices in line with evidence-based recommendations and conducting further research to develop measurement tools to report women’s experiences of abortion care. Delays in seeking care for abortion-related complications increases the risk of severe morbidity, therefore there is a need to strengthen provision of accurate information and services to reduce such delays. It is vital for countries to implement context-specific programmes to increase access to and quality of safe abortion and contraception services.

## References

[1] Ganatra B, Gerdts C, Rossier C, et al Global, regional, and subregional classification of abortions by safety, 2010–14: estimates from a Bayesian hierarchical model. The Lancet 2017;390:2372–81. 10.1016/S0140-6736(17)31794-4 PMC5711001 28964589

[2] Bearak J, Popinchalk A, Ganatra B, et al Unintended pregnancy and abortion by income, region, and the legal status of abortion: estimates from a comprehensive model for 1990–2019. The Lancet Global Health 2020;8:e1152–61. 10.1016/S2214-109X(20)30315-6 32710833

[3] World Health Organization Clinical management of abortion complications: a practical guide. Geneva, Switzerland: World Health Organization, 1994.

[4] World Health Organization Clinical practice Handbook for safe abortion. Geneva, Switzerland: World Health Organization, 2012.

[5] World Health Organization Safe abortion: technical and policy guidance for health systems. Geneva, Switzerland: World Health Organization, 2011.

[6] World Health Organization Medical management of abortion. Geneva, Switzerland: World Health Organization, 2018.

[7] Say L, Chou D, Gemmill A, et al Global causes of maternal death: a who systematic analysis. Lancet Glob Health 2014;2:e323–33. 10.1016/S2214-109X(14)70227-X 25103301

[8] Madziyire MG, Polis CB, Riley T, et al Severity and management of postabortion complications among women in Zimbabwe, 2016: a cross-sectional study. BMJ Open 2018;8:e019658 10.1136/bmjopen-2017-019658 PMC5829940 29440163

[9] Bankole A, Kayembe P, Chae S, et al The severity and management of complications among Postabortion patients treated in Kinshasa health facilities. Int Perspect Sex Reprod Health 2018;44:1–9. 10.1363/44e5618 30138102 PMC6294570

[10] Izugbara C, Wekesah FM, Sebany M, et al Availability, accessibility and utilization of post-abortion care in sub-Saharan Africa: a systematic review. Health Care Women Int 2020;41:732–60. 10.1080/07399332.2019.1703991 31855511

[11] Ziraba AK, Izugbara C, Levandowski BA, et al Unsafe abortion in Kenya: a cross-sectional study of abortion complication severity and associated factors. BMC Pregnancy Childbirth 2015;15:34 10.1186/s12884-015-0459-6 25884662 PMC4338617

[12] Aantjes CJ, Gilmoor A, Syurina EV, et al The status of provision of post abortion care services for women and girls in eastern and southern Africa: a systematic review. Contraception 2018;98:77–88. 10.1016/j.contraception.2018.03.014 29550457

[13] Calvert C, Owolabi OO, Yeung F, et al The magnitude and severity of abortion-related morbidity in settings with limited access to abortion services: a systematic review and meta-regression. BMJ Glob Health 2018;3:e000692 10.1136/bmjgh-2017-000692 PMC6035513 29989078

[14] World Health Organization Standards for improving quality of maternal and newborn care in health facilities. Geneva, Switzerland: World Health Organization, 2016.

[15] Nairobi ICPD 25th summit commitments. Available: http://www.nairobisummiticpd.org/

[16] Kruk ME, Gage AD, Arsenault C, et al High-Quality health systems in the sustainable development goals era: time for a revolution. Lancet Glob Health 2018;6:e1196–252. 10.1016/S2214-109X(18)30386-3 30196093 PMC7734391

[17] Darney BG, Powell B, Andersen K, et al Quality of care and abortion: beyond safety. BMJ Sex Reprod Health 2018;44:159–60. 10.1136/bmjsrh-2018-200060 PMC6225511 29972364

[18] Souza JP, Gülmezoglu AM, Vogel J, et al Moving beyond essential interventions for reduction of maternal mortality (the who multicountry survey on maternal and newborn health): a cross-sectional study. The Lancet 2013;381:1747–55. 10.1016/S0140-6736(13)60686-8 23683641

[19] Souza JP, Cecatti JG, Faundes A Maternal near miss and maternal death in the World Health Organization’s 2005 global survey on maternal and perinatal health. Bulletin of the World Health Organization 2010;88:113–9. 20428368 10.2471/BLT.08.057828 PMC2814475

[20] Souza JP, Gülmezoglu AM, Lumbiganon P, et al Caesarean section without medical indications is associated with an increased risk of adverse short-term maternal outcomes: the 2004-2008 who global survey on maternal and perinatal health. BMC Med 2010;8:71 10.1186/1741-7015-8-71 21067593 PMC2993644

[21] Kim CR, Tunçalp Özge, Ganatra B, et al Who Multi-Country survey on Abortion-related morbidity and mortality in health facilities: study protocol. BMJ Glob Health 2016;1:e000113 10.1136/bmjgh-2016-000113 PMC5321365 28588967

[22] Shah A, Faundes A, Machoki M'Imunya, Machoki M, et al Methodological considerations in implementing the who global survey for monitoring maternal and perinatal health. Bull World Health Organ 2008;86:126–31. 10.2471/BLT.06.039842 18297167 PMC2647388

[23] World Health Organization Monitoring emergency obstetric care – a handbook. Geneva, Switzerland: World Health Organization, 2009.

[24] Royal College of Obstetrics and Gynaecology The management of early pregnancy loss. RCOG Green-top guideline no. 25 2006.

[25] Souza JP, Gülmezoglu AM, Carroli G, et al The world Health organization multicountry survey on maternal and newborn health: study protocol. BMC Health Serv Res 2011;11:286 10.1186/1472-6963-11-286 22029735 PMC3258197

[26] Souza JP, Cecatti JG, Haddad SM, et al The who maternal near-miss approach and the maternal severity index model (MSI): tools for assessing the management of severe maternal morbidity. PLoS One 2012;7:e44129 10.1371/journal.pone.0044129 22952897 PMC3430678

[27] Johnson BR, Lavelanet AF, Schlitt S Global abortion policies database: a new approach to strengthening knowledge on laws, policies, and human rights standards. BMC Int Health Hum Rights 2018;18:35 10.1186/s12914-018-0174-2 30208877 PMC6134502

[28] Rees H, Katzenellenbogen J, Shabodien R The epidemiology of incomplete abortion in South Africa. National incomplete abortion reference group. S Afr Med J 1997;87:432–7. 9254785

[29] World Health Organization Evaluating the quality of care for severe pregnancy complications; the WHO near-miss approach for maternal health. Geneva, Switzerland: World Health Organization, 2011.

[30] Owolabi OO, Cresswell JA, Vwalika B, et al Incidence of abortion-related near-miss complications in Zambia: cross-sectional study in central, Copperbelt and Lusaka provinces. Contraception 2017;95:167–74. 10.1016/j.contraception.2016.08.014 27593334

[31] Kalilani-Phiri L, Gebreselassie H, Levandowski BA, et al The severity of abortion complications in Malawi. Int J Gynaecol Obstet 2015;128:160–4. 10.1016/j.ijgo.2014.08.022 25468057

[32] Dragoman M, Sheldon WR, Qureshi Z, et al Overview of abortion cases with severe maternal outcomes in the who multicountry survey on maternal and newborn health: a descriptive analysis. BJOG: Int J Obstet Gy 2014;121:25–31. 10.1111/1471-0528.12689 24641532

[33] Diamond-Smith N, Percher J, Saxena M, et al Knowledge, provision of information and barriers to high quality medication abortion provision by pharmacists in Uttar Pradesh, India. BMC Health Serv Res 2019;19:476 10.1186/s12913-019-4318-4 31296200 PMC6622002

[34] Pourette D, Mattern C, Ratovoson R, et al Complications with use of misoprostol for abortion in Madagascar: between ease of access and lack of information. Contraception 2018;97:116–21. 10.1016/j.contraception.2017.12.005 29242087

[35] Sedgh G, Bearak J, Singh S, et al Abortion incidence between 1990 and 2014: global, regional, and subregional levels and trends. The Lancet 2016;388:258–67. 10.1016/S0140-6736(16)30380-4 PMC5498988 27179755

[36] Assifi AR, Berger B, Tunçalp Özge, et al Women’s awareness and knowledge of abortion laws: a systematic review. PLoS One 2016;11:e0152224 10.1371/journal.pone.0152224 27010629 PMC4807003

[37] Kaczmarek E How to distinguish medicalization from over-medicalization? Med Health Care Philos 2019;22:119–28. 10.1007/s11019-018-9850-1 29951940 PMC6394498

[38] Sudhinaraset M, Landrian A, Montagu D, et al Is there a difference in women’s experiences of care with medication vs. manual vacuum aspiration abortions? Determinants of person-centered care for abortion services. PLoS One 2019;14:e0225333 10.1371/journal.pone.0225333 31765417 PMC6876888

[39] Cook S, de Kok B, Odland ML 'It’s a very complicated issue here': understanding the limited and declining use of manual vacuum aspiration for postabortion care in Malawi: a qualitative study. Health policy and planning 2017;32:305–13. 27616307 10.1093/heapol/czw128

[40] Kulczycki A The imperative to expand provision, access and use of misoprostol for Post-Abortion care in sub-Saharan Africa. Afr J Reprod Health 2016;20:22–5. 10.29063/ajrh2016/v20i3.3 29553188

[41] Cresswell JA, Schroeder R, Dennis M, et al Women’s knowledge and attitudes surrounding abortion in Zambia: a cross-sectional survey across three provinces. BMJ Open 2016;6:e010076 10.1136/bmjopen-2015-010076 PMC4809085 27000784

[42] Endler M, Lavelanet A, Cleeve A, et al Telemedicine for medical abortion: a systematic review. BJOG: Int J Obstet Gy 2019;126:1094–102. 10.1111/1471-0528.15684 PMC7496179 30869829

[43] World Health Organization Health worker roles in providing safe abortion care and post-abortion contraception. Geneva, Switzerland: World Health Organization, 2015. 26401543

[44] Bohren MA, Hunter EC, Munthe-Kaas HM, et al Facilitators and barriers to facility-based delivery in low- and middle-income countries: a qualitative evidence synthesis. Reprod Health 2014;11:71 10.1186/1742-4755-11-71 25238684 PMC4247708

[45] Bohren MA, Mehrtash H, Fawole B How women are treated during facility-based childbirth in four countries: a cross-sectional study with labour observations and community-based surveys. Lancet 2019. 10.1016/S0140-6736(19)31992-0 PMC6853169 31604660

[46] Sudhinaraset M, Afulani PA, Diamond-Smith N, et al Development of a Person-Centered family planning scale in India and Kenya. Stud Fam Plann 2018;49:237–58. 10.1111/sifp.12069 30069983

[47] Afulani PA, Phillips B, Aborigo RA, et al Person-Centred maternity care in low-income and middle-income countries: analysis of data from Kenya, Ghana, and India. The Lancet Global Health 2019;7:e96–109. 10.1016/S2214-109X(18)30403-0 30554766 PMC6293963

