# Supplementary material for: Understanding abortion-related complications in health facilities: results from WHO multicountry survey on abortion (MCS-A) across 11 sub-Saharan African countries
Source: BMJ Glob Health. 2021 Jan 29;6(1):e003702. doi: 10.1136/bmjgh-2020-003702 (PMC7845704; doi:10.1136/bmjgh-2020-003702)
Supplement: Supplementary data [file bmjgh-2020-003702supp004.pdf]

Understanding abortion-related complications in health facilities: Results from WHO multi-country survey on abortion (MCS-A) across 11 African countries

Supplementary File

3

Annex IV. Criteria for hierarchal classification of abortion-related complications

WHO Near-Miss Criteria (264, 1.9%)		Potentially-life threatening complications (PLTC) (957, 7.0%)		Moderate (7953, 58.2%)		Mild (4424, 32.4%)	
**Organ dysfunction**	**N (%)**	**Symptoms**	**N (%)**	**Symptoms**	**N (%)**	**Signs and symptoms on initial assessment**	**N (%)**
**Cardiovascular**	165 (62.5)	Severe haemorrhage	703 (73.5)	Bleeding *Heavy bright red vaginal bleeding (with or without clots), Blood soaked pads/towels/clothing, pallor*	7656 (96.3)	Vaginal Bleeding	3470 (78.4)
**Respiratory**	61 (23.1)	Severe systemic infection	327 (34.2)	Suspected intraabdominal injury *Abdominal pain/cramping, nausea, vomiting distended/tense/hard abdomen Shoulder pain Decreased bowel sounds, rebound, tenderness*	850 (10.7)	Cervix open	3529 (79.8)
**Renal**	27 (10.2)	Uterine perforation	39 (4.1)	Infection *Chills, fevers, sweats Foul smelling vagina discharge History of interference with pregnancy*	494 (6.2)	Abnormal vital signs *based on temperature, heart rate, systolic/diastolic blood pressure, and respiratory rate*	1156 (26.1)
**Coagulation**	33 (12.5)					Uterine tenderness	736 (16.6)
**Neurologic**	29 (10.9)					Abnormal mental status *Agitated, lethargic, comatose*	627 (14.2)
**Hepatic**	18 (6.8)					Abnormal abdominal examination *Rebounding/guarding. Distended, decreased bowel sounds, tense/hard, tenderness on palpitation*	633 (14.3)
**Uterine**	26 (9.9)					Abnormal appearance *Sick-looking, pallor, jaundice, clammy*	440 (9.9)
						Cervical Motion Tenderness	362 (8.2)
						Foul smelling vaginal discharge	237 (5.4)
						Evidence of foreign body	25 (0.57)
						Adnexal mass	11 (0.25)

References

1. Johnson BR, Jr., Mishra V, Lavelanet AF, Khosla R, Ganatra B. A global database of abortion laws, policies, health standards and guidelines. *Bull World Health Organ* 2017; **95**(7): 542-4. 2.

2. Johnson BR, Lavelanet AF, Schlitt S. Global Abortion Policies Database: a new approach to strengthening knowledge on laws, policies, and human rights standards. *BMC International Health and Human Rights* 2018; **18**(1): 35.
