# Supplementary material for: Understanding abortion-related complications in health facilities: results from WHO multicountry survey on abortion (MCS-A) across 11 sub-Saharan African countries
Source: BMJ Glob Health. 2021 Jan 29;6(1):e003702. doi: 10.1136/bmjgh-2020-003702 (PMC7845704; doi:10.1136/bmjgh-2020-003702)
Supplement: Supplementary data [file bmjgh-2020-003702supp001.pdf]

Understanding abortion-related complications in health facilities: Results from WHO multi-country survey on abortion (MCS-A) across 11 African countries

Supplementary File

Annex I. Development of variables within dataset

Abortion-related national, legal and policy environment
Considering the national, legal and policy environments for provision of safe abortion, a new open-access WHO Global Abortion Policies Database (GAPD) was launched [1]. It contains comprehensive information on the abortion laws, policies, health standards and guidelines for WHO and United Nations (UN) Member States, [1,2] . Using the open-access WHO GAPD, we described the national legal and policy environments surrounding abortion provision across the 11 African countries in our study [2].
We report abortion-related complications by national level characteristics that included grouping countries according to 1) availability of national guidelines (none or post-abortion care only, or induced and post-abortion care guidelines) and, 2) country-recognized essential medicines for medical abortion (none or misoprostol only or misoprostol and mifepristone).

Timing of abortion-related complications based on facility admission
Presentation of abortion-related complication variables were based on clinical admission in the facility. The admission assessment response options were: yes at arrival or within 24 hours of facility stay or yes after 24 hours of facility stay. Less than 0.5% of responses indicated the presentation of the abortion-related complications occurred after 24 hours of facility stay. Therefore, clinical admission indicators for abortion-related complications are interpreted as at arrival/within 24 hours of facility stay for the purposes of the analyses.

Gestational age
There are N=2944 women with an undetermined gestational age. We ran sensitivity analyses to compare women with undetermined gestational age group to the entire sample of women. We assessed sociodemographic, obstetric and clinical characteristics' differences between the entire sample of women and women with undetermined gestational age. Furthermore, we assessed the prevalence of abortion-related complications among the sample of undetermined gestational age. Our analysis illustrated that the two groups were similar and prevalence of abortionrelated complications were also similar to the overall sample of women.

5

References

1. Johnson BR, Jr., Mishra V, Lavelanet AF, Khosla R, Ganatra B. A global database of abortion laws, policies, health standards and guidelines. *Bull World Health Organ* 2017; **95**(7): 542-4. 2.
Johnson BR, Lavelanet AF, Schlitt S. Global Abortion Policies Database: a new approach to strengthening knowledge on laws, policies, and human rights standards. *BMC International Health and Human Rights* 2018; **18**(1): 35.

6
