# Supplementary material for: Understanding abortion-related complications in health facilities: results from WHO multicountry survey on abortion (MCS-A) across 11 sub-Saharan African countries
Source: BMJ Glob Health. 2021 Jan 29;6(1):e003702. doi: 10.1136/bmjgh-2020-003702 (PMC7845704; doi:10.1136/bmjgh-2020-003702)
Supplement: Supplementary data [file bmjgh-2020-003702supp003.pdf]

Understanding abortion-related complications in health facilities: Results from WHO multi-country survey on abortion (MCS-A) across 11 African countries

Supplementary File

Annex III. Facility Characteristics

	Benin	Burkina Faso	Chad	DRC	Ghana	Kenya	Malawi	Mozambique	Niger	Nigeria	Uganda	Total
**Number of participating facilities**	**10**	**21**	**15**	**24**	**19**	**21**	**23**	**19**	**10**	**29**	**19**	**210**
**Location**												
Urban	10	21	14	18	16	18	17	10	10	27	13	**174**
Rural	0	0	1	6	3	3	6	9	0	2	6	**36**
**Facility level**												
Primary	8 (80)	0	1 (6.7)	5 (20.8)	2 (10.5)	0	0	0	0	0	3 (16.7)	**19 (19.0)**
Secondary	0	14 (66.7)	5 (33.3)	16 (66.7)	11 (57.9)	11 (47.9)	22 (95.7)	11 (57.9)	9 (90.)	22 (75.9)	4 (22.2)	**125 (59.2)**
Tertiary	2 (20)	7 (33.3)	8 (53.3)	2 (8.3)	6 (31.6)	8 (34.8)	1 (4.4)	6 (31.6)	0	7 (24.1)	8 (44.4)	**55 (26.1)**
Other	0	0	1 (6.7)	1 (6.7)	0	4 (17.4)	0	2 (10.5)	1 (10.0)	0	3 (16.7)	**12 (5.7)**
**Resources currently used at facility**												
Safe Abortion Guidance/Clinical Handbook	8 (80.0)	17 (80.9)	7 (46.7)	10 (43.5)	14 (73.7)	9 (39.1)	19 (82.6)	14 (73.7)	8 (80.0)	16 (55.2)	8 (42.1)	**130 (62.2)**
WHO guidelines (e.g. for postpartum haemorrhage)	10 (100)	19 (90.5)	8 (53.3)	20 (83.3)	18 (94.7)	20 (95.2)	23 (100)	17 (89.5)	9 (90.0)	26 (89.7)	14 (73.7)	**184 (87.6)**
Evidence-based, locally adapted guidelines	10 (100)	17 (80.9)	5 (33.3)	14 (58.3)	16 (84.2)	17 (80.9)	10 (52.6)	21 (91.3)	8 (80.0)	26 (89.7)	11 (61.1)	**155 (74.2)**
Clinical audits (e.g. maternal deaths reviews)	10 (100)	19 (90.5)	8 (53.3)	22 (91.7)	19 (100)	21 (100)	22 (95.7)	19 (100)	10 (10)	27 (93.1)	15 (78.9)	**192 (91.4)**
**Surgical abortion method employed for gestational age up to 12-14 weeks**	7 (70.0)	18 (85.7)	12 (80.0)	13 (56.5)	14 (73.7)	17 (80.9)	23 (100)	14 (73.7)	9 (90.0)	17 (60.7)	15 (78.9)	**159 (76.4)**
**Medical abortion offered at the facility (including medical management of incomplete abortion)**	10 (100)	19 (90.5)	14 (93.3)	12 (54.6)	14 (73.7)	18 (85.7)	19 (82.6)	11 (57.9)	8 (80.0)	27 (93.1)	16 (84.2)	**168 (80.8)**
**Abortion for gestational age >13 weeks offered at facility**	7 (70.0)	18 (85.7)	13 (86.7)	10 (45.5)	10 (52.6)	14 (66.7)	20 (86.9)	8 (42.1)	6 (60.0)	18 (62.1)	7 (36.8)	**131 (62.9)**
**Post-abortion contraception offered at facility**	9 (90.0)	19 (90.5)	14 (93.3)	18 (75.0)	18 (94.7)	19 (90.5)	22 (95.7)	19 (100)	10 (100)	28 (96.6)	14 (73.7)	**190 (90.5)**

References

1. Johnson BR, Jr., Mishra V, Lavelanet AF, Khosla R, Ganatra B. A global database of abortion laws, policies, health standards and guidelines. *Bull World Health Organ* 2017; **95**(7): 542-4.
2. Johnson BR, Lavelanet AF, Schlitt S. Global Abortion Policies Database: a new approach to strengthening knowledge on laws, policies, and human rights standards. *BMC International Health and Human Rights* 2018; **18**(1): 35.
