# Supplementary material for: Understanding abortion-related complications in health facilities: results from WHO multicountry survey on abortion (MCS-A) across 11 sub-Saharan African countries
Source: BMJ Glob Health. 2021 Jan 29;6(1):e003702. doi: 10.1136/bmjgh-2020-003702 (PMC7845704; doi:10.1136/bmjgh-2020-003702)
Supplement: Supplementary data [file bmjgh-2020-003702supp002.pdf]

Understanding abortion-related complications in health facilities: Results from WHO multi-country survey on abortion (MCS-A) across 11 African countries

Supplementary File

Annex II. Legal status of abortion among 11 participating African countries[1]

Legal Classification	Country
Law prohibits all abortion	None
Unlawful abortion is prohibited or where there are only penalties for unlawful abortion, with no additional information provided about lawful abortion	Nigeria
Law allows or permits abortion only on one or more legal grounds	Benin, Burkina Faso, Congo, Chad, Ghana, Kenya, Malawi, Niger, Uganda
Law entitles a woman to abortion on request with no requirement for justification	Mozambique

[1] Johnson, B.R., Lavelanet, A.F. & Schlitt, S. Global Abortion Policies Database: a new approach to strengthening knowledge on laws, policies, and human rights standards. BMC Int Health Hum Rights 18, 35 (2018) doi:10.1186/s12914-018-0174-2

References

1. Johnson BR, Jr., Mishra V, Lavelanet AF, Khosla R, Ganatra B. A global database of abortion laws, policies, health standards and guidelines. *Bull World Health Organ* 2017; **95**(7): 542-4. 2. Johnson BR, Lavelanet AF, Schlitt S. Global Abortion Policies Database: a new approach to strengthening knowledge on laws, policies, and human rights standards. *BMC International Health and Human Rights* 2018; **18**(1): 35.
