# Supplementary material for: Understanding abortion-related complications in health facilities: results from WHO multicountry survey on abortion (MCS-A) across 11 sub-Saharan African countries
Source: BMJ Glob Health. 2021 Jan 29;6(1):e003702. doi: 10.1136/bmjgh-2020-003702 (PMC7845704; doi:10.1136/bmjgh-2020-003702)
Supplement: Supplementary data [file bmjgh-2020-003702supp005.pdf]

Understanding abortion-related complications in health facilities: Results from WHO multi-country survey on abortion (MCS-A) across 11 African countries

Supplementary File

Annex V. National, sociodemographic and obstetric characteristics of ACASI study population by severity of abortion-related complications

	Entire population (N=13657)	ACASI (N=3091)
**SOCIODEMOGRAPHIC AND OBSTETRIC**		
**Age (in years)**	**3051**	**13516**
≤19	490 (16.1)	2173 (16.1)
20 - 29	1419 (46.5)	6565 (48.6)
≥30	1142 (37.4)	132 (41.7)
**Marital Status**	**2966**	**12614**
Single	651 (21.9)	2852 (22.6)
Other than single	2315 (78.1)	9762 (77.4)
**Education**	**2628**	**10485**
No Education	437 (16.6)	1791 (17.1)
Some education	2191 (83.4)	8694 (82.9)
**Gainful occupation**	**2750**	**11708**
Yes	1336 (48.6)	5276 (45.1)
**Previous pregnancies**	**3023**	**13264**
0	816 (26.9)	3584 (27.0)
1 or more	2207 (73.0)	9680 (72.9)
**Previous abortions**		
0	1321 (61.3)	6050 (64.1)
1 or more	833 (38.7)	3384 (35.9)
**Gestational age (in weeks)***		
<13	1707 (55.2)	7716 (56.5)
13-28	899 (29.1)	3496 (25.6)
Undetermined	485 (15.7)	2445 (17.9)
**Expulsion of products of conception before arrival**	**3081**	**13616**
Yes	1327 (43.1)	5916 (43.5)

*p-value <0.0001

References

1. Johnson BR, Jr., Mishra V, Lavelanet AF, Khosla R, Ganatra B. A global database of abortion laws, policies, health standards and guidelines. *Bull World Health Organ* 2017; **95**(7): 542-4. 2. Johnson BR, Lavelanet AF, Schlitt S. Global Abortion Policies Database: a new approach to strengthening knowledge on laws, policies, and human rights standards. *BMC International Health and Human Rights* 2018; **18**(1): 35.
